# Well Formed Children

**Published:** 1890-03

**Authors:** 


### Editorial, Etc.

WELL-FORMED CHILDREN.—SCHOOL TEACHERS SAY THEY ARE VERY LARGELY IN THE MINORITY.—The Boston Herald says: "The common school teacher finds perfectly healthy children are a rarity. Seldom will ten per cent. of her class of fifty be found free from any physical defect whatever and with true development for their age. Of these it must be regretfully admitted that the smallest number are Americans. The best formed school children and the healthiest are the Germans and Bohemians, the next of Irish parentage. Nowhere, as in the south and west of Ireland, where children run free of care the year round, hatless, shoeless, living on the coarsest, plainest food, and yet, with some measure of school restrictions, can such specimens of perfect physical beauty in form and face be found. Glance over any school-room, with its many upturned faces inquisitively questioning the thought of the observer, and but few really fine-featured children are to be found after the age of ten is passed.

When a handsome face, answering all the laws that govern beauty, is discovered, it is invariably synonymous with good health in its possessor, and all too often of foreign parentage or birth. For these two defects, the want of good health and good looks and the added one of grace, in the majority of public school children, one is naturally led to think of a cause, but instead of one there are many. The primary one may be found in the following remark of Frances Willard: "Women's everlasting befrilled, bedizened and bedragged style of dress is to-day doing more harm to children unborn, born and dying than all other causes that compel public attention."

Class recitations where pupils stand are now in most schools done away with, and this is fortunate, since pupils were sometimes kept an unconscionably long time on the floor, resulting in evils one dreads to think of, and unfortunate in that the children have all the session long no change of position. But the teacher who insists on having her troublesome, uneasy little fellow stand for a half hour has much to answer for, as well as one who argues strongly on the abolition of the recess. One cannot but be sympathetic with the teacher. If she does not keep her pupils like so many trained dummies she is a poor disciplinarian and subject to dismissal. If her class does not know every date from Adam to Harrison, and the location of all cross road towns from Siberia to Patagonia, she is not keeping up in that modern bugbear, the "course of study."

Dr. Anderson gives a list of the most common physical defects found in pupils in the public schools. They are summarized as—

Head—Droops forward; carried a little to one side, chin raised too high.

Shoulders—Round, sloping, stooping and uneven; one lower than the other.

Thorax—One side better developed than the other; the diameter at the base too short.

Upper Back—Right shoulder blade too prominent in right-handed people.

Spine—Bends too far forward from between the shoulders.

Waist—Too narrow; abdominal muscles weak.

Hips—Thrown too far forward.

Arms—Forearm better developed than the upper arm.

Legs—Better developed than thigh.

Thigh—Inside and back poorly developed.

Any person entering the examining room of a gymnasium for boys would be astonished at the large numbers that have one or even more of these defects, and with girls this is found almost universal. While with boys indulging in frequent play there will be found good muscular developement of the lower limbs, the trunk, that part holding the vital organs, will but occasionally be found perfect on examination. As everyone knows, poor circulation is a common fault, and can be remedied by exercise properly taken.

---

### Bibliographical.

